# Lanthanum Gadolinium Oxide: A New Electronic Device Material for CMOS Logic and Memory Devices

**DOI:** 10.3390/ma7042669

**Published:** 2014-03-31

**Authors:** Shojan P. Pavunny, James F. Scott, Ram S. Katiyar

**Affiliations:** 1 Department of Physics and Institute for Functional Nanomaterials, University of Puerto Rico, P.O. Box 70377, San Juan, PR 00936-8377, USA; E-Mail: jfs32@cam.ac.uk; 2 Department of Physics, Cavendish Laboratory, University of Cambridge, Cambridge CB3 OHE, UK

**Keywords:** LaGdO_3_, gate oxide, high-k dielectrics, amorphous, optical, metal-oxide-semiconductor, metal-insulator-metal, equivalent oxide thickness

## Abstract

A comprehensive study on the ternary dielectric, LaGdO_3_, synthesized and qualified in our laboratory as a novel high-k dielectric material for logic and memory device applications in terms of its excellent features that include a high linear dielectric constant (k) of ~22 and a large energy bandgap of ~5.6 eV, resulting in sufficient electron and hole band offsets of ~2.57 eV and ~1.91 eV, respectively, on silicon, good thermal stability with Si and lower gate leakage current densities within the International Technology Roadmap for Semiconductors (ITRS) specified limits at the sub-nanometer electrical functional thickness level, which are desirable for advanced complementary metal-oxide-semiconductor (CMOS), bipolar (Bi) and BiCMOS chips applications, is presented in this review article.

## 1. Introduction

The integration of silicon-based metal-oxide-semiconductor field-effect-transistors (MOSFETs) still fulfill Moore’s law [1] by delivering microprocessors with Dennard-scaled dimensions, better data processing speed and higher energy efficiency (low power consumption) from one technology node to another. As the field effect transistors migrate from one generation to the other, the gate lengths are decreased and the spacing between the key contacts—gate, source and drain—reduces to a few tens of nanometers effecting the overlap of the electric fields that they generate. The resulting short channel effect is known as drain-induced barrier lowering (DIBL) [2]. The extent to which the drain has control in the channel is termed the natural length (λ_l_) of the device, a parameter that is to be kept at 0.2 to 0.1 times the gate length in the device design in order to have well-controlled short channel characteristics. For a planar single-gate transistor, this term is formulated as:

$$\lambda_{1}=\sqrt{t_{\mathrm{Si}}t_{\text{GO}}\frac{\varepsilon_{\mathrm{Si}}}{\varepsilon_{\text{GO}}}}$$ (1)

where *t*_Si_ and *t*_GO_ are the thicknesses of the silicon and gate oxide layer and ε_Si_ and ε_GO_ are their permittivity. It is clear from Equation (1) that density scaling in future logic generations with minimized short channel effects (or natural length λ_l_) can be realized by thinning down the thicknesses of the channel layer (the concept of ultra-thin-body fully depleted silicon-on-insulator (UTB FD SOI) CMOS) and the gate oxide layer and by using a qualified high-k gate dielectric of higher permittivity than SiO_2_- or hafnium silicate-based gate oxide materials.

An intensive quest for alternative high-k gate oxides dictated by International Technology Roadmap for Semiconductors (ITRS) [3] commenced in the 1990s for enhanced capacitance density or electrostatic control and reduced gate oxide leakage or standby power. However, it took more than a decade for its implementation into the mainstream CMOS process flow. In 2007, high-k/metal-gate (HKMG) technology was successfully launched in the 45-nm logic technology generation to address the drawbacks of high-k/poly-Si structures, such as higher threshold voltages due to Fermi-level pinning at the interface [4] and severely degraded channel mobility caused by the coupling of the low energy surface optical phonon modes (arising from the polarization of the high-k dielectric) to the inversion channel charge carriers [5]. This entry of HKMG marked a revolutionary change in transistor technology in terms of lower gate leakage, lower switching power and higher drive current. Afterwards, non-traditional scaling methodologies, like multi-gate transistors (finFETs), fully depleted silicon-on-insulator (FDSOI) and strained silicon, helped in the continued scaling down to the 22-nm node.

Metal-insulator-metal (MIM) stacked capacitors find extensive applications, such as radiofrequency (RF) coupling and bypass capacitors in oscillator and resonator circuits, filter and analog capacitors in analog/mixed-signal (AMS) circuits, decoupling capacitors for microprocessors (MPUs), as well as storage capacitors in dynamic random access memory (DRAM) and embedded DRAM (eDRAM)/logic devices. The efficient approach towards density scaling MIM devices is to replace the conventional silicon dioxide (SiO_2_) or silicon nitride (Si_3_N_4_) films by alternative high-k stacked charge storage layers for higher capacitance density (>20 fF/μm^2^), reduced feature size (4f^2^ cell size, where 4 is a design factor and f the half-pitch in microns), low standby power (~10^−8^ A/cm^2^ at 1 V) at the access transistor and storage capacitor and stable sensing (good contrast in logic levels). The new dielectric material must fulfill additional requirements, such as good capacitance (permittivity)-voltage linearity, a high quality factor (*Q* = (1/tan δ) > 20) and an elevated breakdown voltage.

As required in the ITRS process integration, devices and structures (PIDS) table, targeted equivalent oxide thicknesses (EOTs) of ~7.3 Å and ~3 Å were needed for alternative high-k dielectric materials as early as 2014 in the 18-nm logic and 24-nm DRAM technology generations, respectively. Such a lower electrical functional thickness may be unfeasible in logic devices with the materials used at present, due to their relatively low dielectric constant ≤15. This situation requires that new candidate materials be identified soon. To this end, we have developed a promising inter-lanthanide oxide-based high-k material, LaGdO_3_, and characterized it as a potential candidate for capacitors for future sub-nanometer logic (MOSFETs) and memory nodes (DRAM and MIM films). In this article, we present a comprehensive review of the structural, optical, dielectric and electrical properties of this new electronic device material and demonstrate it as a material of interest/importance [6].

## 2. Theoretical Estimation of k-Value of LaGdO_3_

The theoretical relationship between the dielectric polarizability and k-value (ε_r_) of a material is given by the well-known Clausius–Mossotti equation [7]:

$$\varepsilon_{r}=\frac{\left(3V_{m}+8{\pi \alpha }_{D}^{T}\right)}{\left(3V_{m}-4{\pi \alpha }_{D}^{T}\right)}$$ (2)

where *V*_m_ and $\alpha_{D}^{T}$ are the molar volume and total dielectric polarizability of the material, respectively. The molar volume is formulated as:

$$V_{m}=\frac{abc\sqrt{1-\cos^{2}\alpha -\cos^{2}\beta -\cos^{2}\gamma +2\cos\alpha \cdot \cos\beta \cdot \cos\gamma }}{Z}$$ (3)

where *a*, *b* and *c* are unit cell axes lengths, α, β and γ are the inter-axial angles and *Z* is the number of formula units per unit cell. The molar volume was calculated as 78.31 Å^3^ using LaGdO_3_ lattice parameters reported by Wenhui *et al.* [8] and coordination number 6. The total dielectric polarizability (16.47 Å^3^) of this multicomponent oxide was calculated by employing La^+3^, Gd^+3^ and O^−2^ polarizabilities (6.07 Å^3^, 4.37 Å^3^ and 2.01 Å^3^ respectively) from Shannon’s list [9] and the additivity rule, which defines that the total dielectric polarizability is the sum of the individual dielectric polarizabilities, α*_D_*(*i*), of all the ions in the formula unit.

$$\alpha_{D}^{T}=\sum_{i=1}^{n}v_{i}\alpha_{D}(i)$$ (4)

where *n* is the total number of types of ions in the formula unit and *v_i_* is the number of Type *i* in the formula unit. The relative dielectric permittivity (ε_r_) value of the LaGdO_3_ was estimated to be 23.1. Such a higher dielectric constant of this new electronic device material is of great interest for the semiconductor industry.

## 3. Qualification of LaGdO_3_ As a Potential High-k Dielectric

In this section, synthesis of LaGdO_3_ dielectric ceramics and identification of this compound as a potential high-k material through their structural, chemical, dielectric and electric characterizations will be discussed.

### 3.1. Synthesis, Phase Identification and Structural Properties of LaGdO_3_ Ceramics

Polycrystalline LaGdO_3_ powders were synthesized by a high-energy solid state reaction from a stoichiometric mixture of La_2_O_3_ (99.9%) and Gd_2_O_3_ (99.9%) precursors that were pre-fired at 700 °C in argon atmosphere to remove the water content. Mixtures of La_2_O_3_ and Gd_2_O_3_ powders were mechanically ball milled for 10 h and then calcined at various temperatures between 1100 to 1500 °C for 12 h. The phase purity of the material was checked by X-ray diffractometry (XRD). The as-synthesized single phase powders were pressed in the form of a thick pellet at four tons of pressure and later sintered at 1500 °C for 4 h. Rietveld refinement of XRD spectra and damped harmonic oscillator modeling of the Raman spectra were employed to establish the layered B-type monoclinic crystal structure of LaGdO_3_ and to determine the atomic positions, coordination numbers, inter-ionic lengths, *etc.*

**Figure 1.**
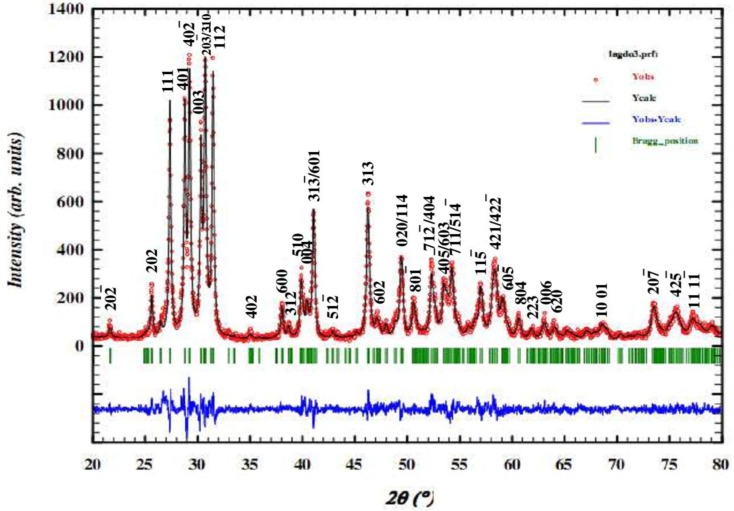
The experimental and pseudo-Voigt modeled diffraction patterns of LaGdO_3_ along with peak indices/index data after final Rietveld least squares minimization. Reprinted with permission from [11]. Copyright 2014 WILEY-VCH Verlag GmbH & Co. KGaA, Weinheim.

Figure 1 shows the results of XRD analysis of the bulk material and reveals the formation of the single phase with a B-type monoclinic structure and a *C*_2_*/m* space group [10]. The unit cell parameters measured at ambient temperature were *a* = 14.4331Å, *b* = 3.6888Å, *c* = 8.9967Å and β = 100.572° and are in good agreement with those reported elsewhere [8]. One may note that the incorporation of larger isovalent La ions into Gd_2_O_3_ (JCPDS (Joint Committee on Powder Diffraction Standard) # 42-1465) resulted in an increase in the lattice parameters and, hence, the unit cell volume. Employing these refined (larger) cell parameters in a Clausius–Mossotti relationship, the re-calculated relative dielectric permittivity (ε_r_) of LaGdO_3_ was 22.69, slightly lower than the value obtained in the previous section due to enhanced molar volume *V*_m_.

**Figure 2.**
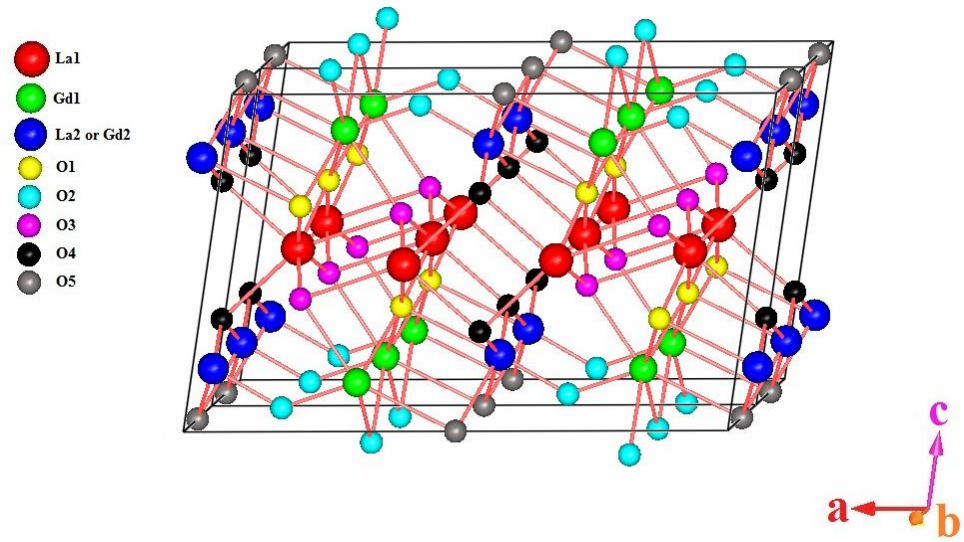
A three-dimensional sketch of the refined B-type monoclinic structure. Reprinted with permission from [11]. Copyright 2014 WILEY-VCH Verlag GmbH & Co. KGaA, Weinheim.

Figure 2 shows the three-dimensional perfectly layered structure model of a large B-type LaGdO_3_ unit cell projected along the *b*-axis. Consecutive layers are separated by *b*/2 along the *a*- and *c*-axes, and they are stacked along the *b*-axis. At ambient conditions, the unit cell consists of two primitive cells, six formula units and 30 atoms. The 12 cations (6 La and 6 Gd ions) having a six or seven oxygen coordination number are seated at centro-symmetric sites with *m*/*C*_s_ symmetry crystallographic sites.

**Figure 3.**
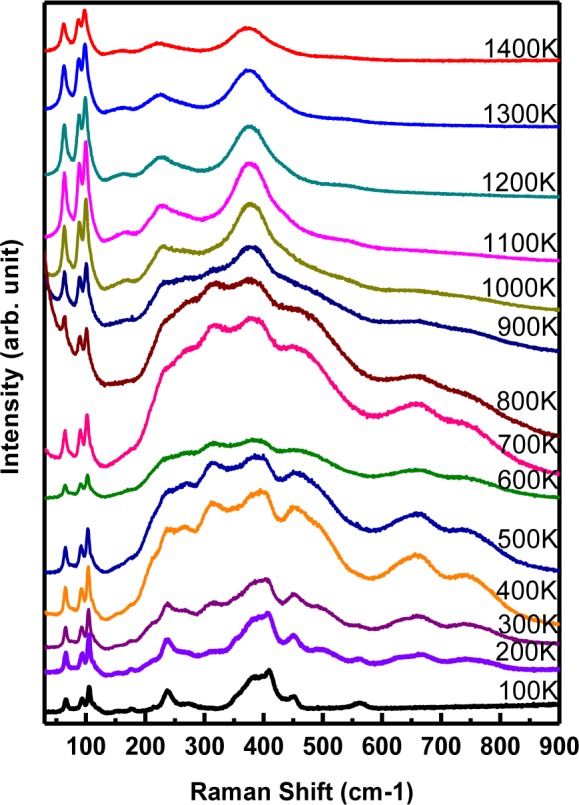
Thermal evolution of the Raman spectra of LaGdO_3_ ceramics from 100 K to 1400 K, with 10 mW of power at a 514.5-nm laser wavelength, an exposure time of 30 s and at least three recordings of each spectrum. Reprinted with permission from [11]. Copyright 2014 WILEY-VCH Verlag GmbH & Co. KGaA, Weinheim.

Figure 3 demonstrates the temperature-dependent behavior of all observed Raman active modes of LaGdO_3_ bulk from 100 K to 1400 K at an interval of 100 K. The factor group analysis for the B-type monoclinic structure is given by Γ*_opt_* = 14*A_g_* (*r*) + 7*B_g_*(*r*) 7*A_u_*(*ir*) 14 *B_u_*(*ir*) [12], where Γ_opt_ are the optical vibrational modes and the symbols, *r* and *ir*, indicate Raman and infrared active modes, respectively.

The analysis of the observed vibrational modes of LaGdO_3_ ceramics were carried out using the damped harmonic oscillator (DHO) model [13]. Out of the predicted 21 Raman-active modes, 17 modes were distinct at 300 K and well fitted with the anharmonic phonon function. The temperature-dependent Raman spectra demonstrates that: (i) all of the low frequency modes below 120 cm^−1^ were almost temperature independent (softening within 3–5 cm^−1^) and intense until 1400 K; (ii) in comparison with the low frequency modes, other modes were more intense near the phase transition temperature (600–900 K); (iii) the 230–240 cm^−1^ mode was very intense below 500 K, and it became a sharp shoulder until 900 K with the evolution of several other intense modes between 200 and 450 cm^−1^; (iv) most of the mid-frequency modes disappeared above 900 K; (v) only two sharp modes were present in the mid-frequency range; and (vi) three diffuse luminescence peaks at 637, 671.1 and 753.9 cm^−1^ disappeared below 160 (±5 K) and above 1000 K. Above 900 K, the disappearance of all high frequency modes (>600 cm^−1^), the merger of several mid-frequency modes (between 200 and 500 cm^−1^) into two distinct modes and the persistence of a total of seven well-defined modes was observed, a direct indication of a possible structural phase transition from monoclinic to tetragonal/pseudocubic.

Energy-dispersive X-ray (EDX) and X-ray fluorescence (XRF) analysis confirmed that the annealed/sintered ceramics are stoichiometric with a La_2_O_3_:Gd_2_O_3_ mass percentage ratio of 48:52 (not shown). X-ray photoelectron spectroscopy (XPS) was employed to study the elemental composition, empirical formula, chemical state and electronic state (+3) of the elements that exist in the compound [14].

### 3.2. Dielectric Properties and Electrical Conduction of LaGdO_3_ Ceramics

**Figure 4.**
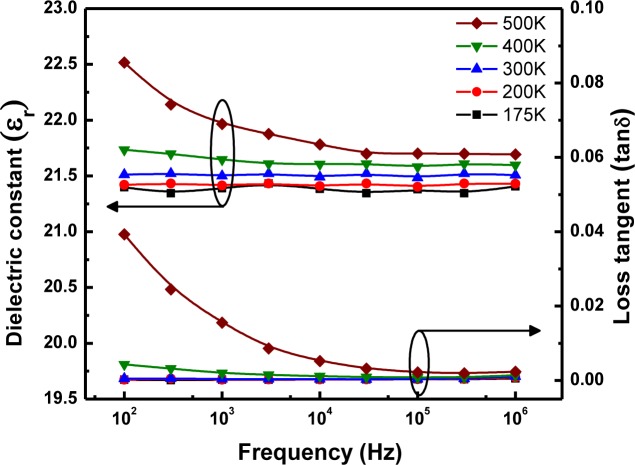
Variation in the dielectric constant and loss tangent of LaGdO_3_ ceramics as a function of frequency in the 175–500 K temperature range. Reprinted with permission from [15]. Copyright 2012 AIP Publishing LLC.

For temperature-dependent dielectric and DC leakage current measurements, LaGdO_3_ pellets of 1 mm in thickness and 7 mm in diameter were DC sputtered at room temperature with Pt to form the top and bottom electrodes, and the resulting MIM structures were annealed at 400 °C in air for proper adhesion of Pt and the recovery of the possible sputter damage. Figure 4 depicts the variation in the real part of the dielectric permittivity (ε_r_) and loss tangent (tanδ) as a function of the frequency (100 Hz to 1 MHz) in the temperature range of 175 K to 500 K. The k-value and loss tangent were found to be ~21.5 and ~0.003, respectively, at 300 K. The variation in ε_r_ is less significant (+4% and −1%), and the loss tangent is less than 0.005 throughout the temperature and frequency measurement range. These features are advantageous as far as the gate oxide and DRAM applications of this material are concerned. Furthermore, one may note that the experimentally achieved value of ε_r_ is comparable with the theoretical prediction given in the previous section.

**Figure 5.**
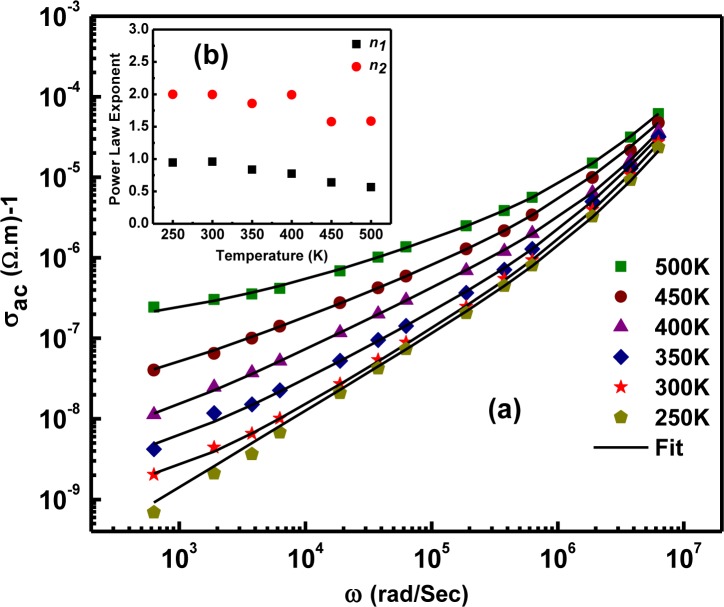
(**a**) AC conductivity (σ_ac_) as a function of frequency in the 250–500 K temperature range along with the double power law fit; (**b**) the variation of the double power law exponents (*n*_1_, *n*_2_) with temperature. Reprinted with permission from [15]. Copyright 2012 AIP Publishing LLC.

Figure 5a shows an increase in AC conductivity (σ_ac_) with frequency for various temperatures. The frequency dependence of the dynamic conductivity follows the double power law with a long-range translational hopping-based DC plateau (Region I, below 3 kHz), a short-range hopping based mid-frequency region (Region II, 3–100 kHz) and a localized or reorientational hopping-based high frequency region (Region III, 100–1000 kHz). The dispersive behavior in σ_ac_ is found to obey the double power law given by:

$$\sigma_{\text{ac}}=\sigma_{\text{dc}}+A\omega^{n_{1}}+B\omega^{n_{2}};0<n_{1}<1,\;1<n_{2}<2$$ (5)

where σ_dc_ is the frequency-independent DC conductivity, ω = 2πf is the angular frequency and *A*, *B*, *n*_1_ and *n*_2_ are material-dependent constants. The parameters evaluated after simulation are given in Table 1 as a function of temperature. Throughout the range, the double power law exponents show an overall decreasing trend with temperature (Figure 5b), indicative of a large polaron hopping mechanism.

**Figure 6.**
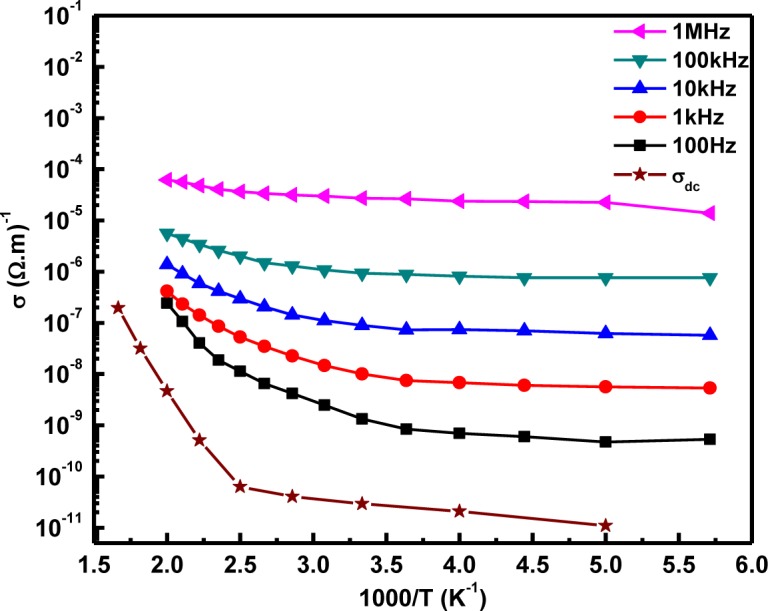
The AC and DC electrical conductivity of LaGdO_3_ as a function of reciprocal temperature. Reprinted with permission from [15]. Copyright 2012 AIP Publishing LLC.

Figure 6 shows the electrical conductivity (log scale) plots as the function of 1000/T (K^−1^) for DC, 100 Hz, 1 kHz, 10 kHz, 100 kHz and 1 MHz. σ_dc_ at room temperature is 2–6 orders less than σ_ac_, since the electrons confined to traps can contribute to alternating current, not to steady current. The dynamic conductivity increases with the increase in temperature and approaches DC conductivity asymptotically at all frequencies at elevated temperatures. The DC conductivity verified the validity of the Arrhenius relation. A very low activation energy of 0.05 eV in the 200–400 K range suggests that electronic charge carriers are predominant in the electrical conduction, whereas the high activation energy of 0.92 eV in the 400–600 K range advocates for the mobility of oxygen ions.

**Figure 7.**
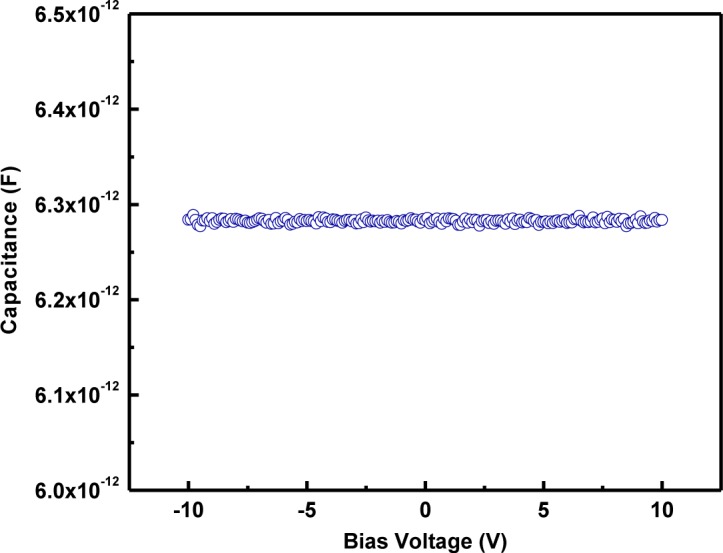
Capacitance (permittivity)-voltage linearity of the Pt/LaGdO_3_/Pt metal-insulator-metal (MIM) structure at 1 MHz. Reprinted with permission from [15]. Copyright 2012 AIP Publishing LLC.

Figure 7 shows the capacitance-voltage (*C-V*) characteristics of LaGdO_3_ ceramics in the MIM configuration at 1 MHz. There is little variation in capacitance with the application of bias voltage. No hysteresis (linear electrical response) was obtained while sweeping the bias voltage from −10 V to +10 V.

**Figure 8.**
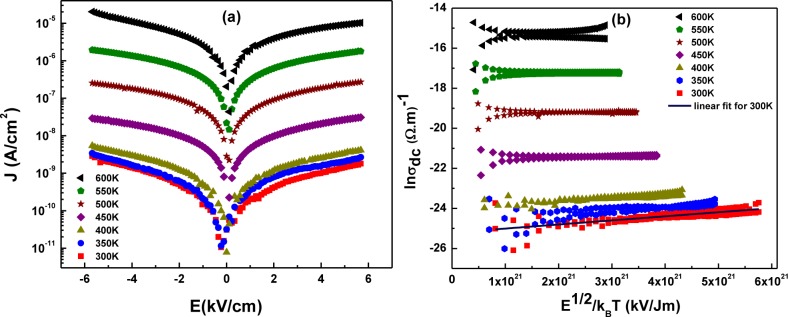
(**a**) Temperature dependency of leakage current density of the Pt/LaGdO_3_ (bulk)/Pt MIM capacitor; (**b**) the Poole–Frenkel plot of the temperature dependence of the DC conductivity at various applied electric fields. Reprinted with permission from [15]. Copyright 2012 AIP Publishing LLC.

Figure 8a shows the symmetric J-E (leakage current density *versus* applied electric field) plots of LaGdO_3_ bulk with respect to positive and negative bias. The leakage current density measured of the MIM stack was very low (2 nA/cm^2^ at 5.7 kV/cm) at 300 K and increased with temperature up to 20 μA/cm^2^ at 5.7 kV/cm at 600 K. Figure 8b shows the linear lnσ_dc_ *versus E*^1/2^/k_B_*T* plot for negative and positive voltages. The optical dielectric constant, ε_∞_, estimated from the slope of the plot at room temperature is 3.6 and 0.9 for Poole–Frenkel (PF) and Schottky emission (SE) [16], respectively. In this case, SE can be ruled out, as ε_∞_, cannot be less than one (or the velocity of light cannot be greater than c in the insulator), and one can conclude that the bulk limited PF may be the major electrical transport mechanism. We have shown in earlier work [17] that the conduction is not due to Simmons modification of the Schottky emission. The Simmons model is often correct for conduction in oxides where the electron mean-free path is smaller than the Schottky barrier width; but in the present case, the fact that Poole–Frenkel models yield the correct optical dielectric constant is an unambiguous proof of that mechanism.

LaGdO_3_ has been identified as a potential high-k candidate for the future CMOS and DRAM technology generations, in terms of its electrical properties in the ceramic form. Hence, LaGdO_3_ thin films deposited on suitable substrates were investigated for such applications, and the results are presented in the following sections.

## 4. Optical Properties of Amorphous LaGdO_3_ Thin Films and Their Electronic Band Match-Up with Si

A high-energy bandgap and a high band offset with the channel (Si in this case) are two of the major criteria in selecting a new high-k material that can be used as a gate dielectric in logic devices or as the bottom layer of a dielectric stack in a memory device. For reliable device performance, the leakage currents can be decreased exponentially by using materials with higher conduction and valence band discontinuities (preferably >1.5 eV) [18]. Optical constants, such as the energy bandgap (*E*_g_), the refractive index (*n*), the extinction coefficient (*k*), the long wavelength refractive index (*n*_∞_) *etc.*, can be determined accurately by carrying out optical characterization, which, in turn, helps in deriving the degree of disorder, electronic band structure and defect concentrations. The carrier transport properties at a high-k oxide/semiconductor interface can be well inferred by deducing the electron affinity (χ), and charge neutrality level (Φ_CNL_) in addition to *E*_g_. Accurate knowledge of these optical and electronic parameters is essential for identifying potential device applications of high-k dielectrics in microelectronics, optoelectronics and optics. Optical dielectric functions and the band lineup of LaGdO_3_ thin films fabricated on quartz and HF (hydrofluoric acid)-last pre-cleaned bare silicon substrates by pulsed laser deposition (PLD) were determined by analyzing the UV/visible transmission, and XPS spectra along with those of RHEED (reflection high energy electron diffraction) and XRD, are presented in this section.

**Figure 9.**
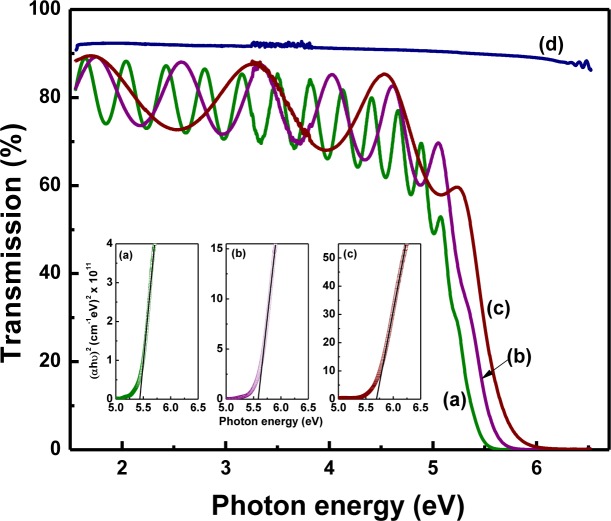
Optical transmittance spectra at normal incidence of amorphous LaGdO_3_ thin films on quartz substrates of (**a**) 725 nm; (**b**) 350 nm; (**c**) 170 nm; and (**d**) quartz substrate. The inset shows the corresponding Tauc’s plots for the three LaGdO_3_ samples, (**a**) 725 nm; (**b**) 350 nm and (**c**) 170 nm, to determine the energy bandgap. Reprinted with permission from [19]. Copyright 2012 AIP Publishing LLC.

Figure 9 shows the well-oscillating transmittance spectra of LaGdO_3_ thin layers with ~90% transmission and a clear absorption edge. The direct bandgap (*E*_g_) was determined from the modified square law-based bandgap calculations using (*αhν*)^2^ *versus hν* plots, by extrapolating the linear portion of the absorption curve (linear fit) to the *x*-axis, where absorption coefficient becomes zero (Figure 9, inset). The absorption edge shifts towards higher energy with the decrease in film thickness, and that resulted in the *E*_g_ increase (blue shift, Δ*E*_g_ 0.27 eV). The measured high *E*_g_ values are 5.43, 5.6 and 5.7 eV for films with 725-nm, 350-nm and 170-nm thicknesses, respectively. A higher degree of amorphousness and defect concentration may be the reason for the observed blue shift in *E*_g_ in thinner films [19].

**Figure 10.**
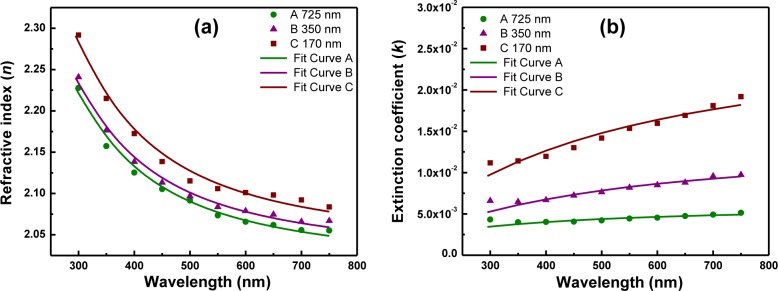
The refractive index spectral density (**a**,**b**) of the LaGdO_3_ thin films determined using the envelope method. Cauchy’s polynomials fit well in the 300–750 nm range, indicative of the applicability of the Cauchy–Urbach model. Reproduced by permission of ECS–The Electrochemical Society [24].

The optical constants, refractive index (*n*) and extinction coefficient (*k*) of all the films have been estimated from the optical transmission spectra by using the “envelope method” [20], and the data are illustrated in Figure 10 along with their Cauchy–Urbach modeling [21]. Substituting the simulated parameters, the dispersion model for LaGdO_3_ can be formulated as:

$$n(\lambda )=2.05+\frac{8.76\times 10^{3}}{\lambda^{2}}+\frac{7.98\times 10^{8}}{\lambda^{4}}$$ (6)

$$k(\lambda )=5.82\times 10^{-3}\exp-1.9\times 10^{-2}\left(12400\left(\frac{1}{\lambda }-\frac{1}{300}\right)\right)$$ (7)

Further, the refractive index dispersion behavior was found to have a linear fit showing the applicability of the Sellmeier single electronic oscillator model [19,22], and the important physical parameters evaluated for LaGdO_3_ are summarized in Table 2. The values of the refractive index estimated were high and in the 2.05–2.29 range. The total optical losses caused by absorption and scattering *k*-values were very low (4 × 10^−3^–17.5 × 10^−3^) for the high-quality films. One may note that the values of the long wavelength refractive index, *n*_∞_, calculated were in the range of 2.02–2.05 and are compatible with the value (1.9) estimated from the leakage conduction studies in LaGdO_3_ ceramics in the previous section. The electronic dielectric constant ${(\varepsilon }_{\infty }=n_{\infty }^{2})$, which is termed the dielectric responses of valence electrons, was derived for the three LaGdO_3_ samples and were 4.104, 4.124 and 4.2, in the order of decreasing thickness. This parameter is small in comparison with the dielectric constant (ε_r_ = ε_∞_ + ε_1_) measured for LaGdO_3_ ceramics ~21.5. In other words, this ternary oxide has a lattice dielectric constant (ε_l_, the dielectric responses of lattice vibrations) much bigger than its electronic counterpart.

One of the basic criteria to select an alternative high-k dielectric material is that it must form a high quality interface with the Si channel. The interface behavior of a given high-k material can be understood by a key parameter, called pinning factor *S*, independent of the metal or semiconductor with which it forms an interface at a metal-semiconductor interface (MSI) or oxide-semiconductor interface (OSI). The factor, *S*, a dimensionless quantity, is a measure of the degree of alignment or pinning caused by the interface states at an insulator (wide energy gap semiconductor)/semiconductor heterostructure and is given by the Monch empirical relation [23].

$$S=\frac{1}{1+0.1{\left(n_{\infty }^{2}-1\right)}^{2}}$$ (8)

The value of *S* falls in the range of zero to one. A value of “0” corresponds to a strong pinning or Bardeen limit, and a value “1” indicates the unpinned Schottky limit. The pinning factor for LaGdO_3_ films was estimated by substituting the long wavelength refractive index, *n*_∞_, values in Equation (8) and was found to follow the theoretical variation in *S* with the electronic component of dielectric constant ε_∞_ or $n_{\infty }^{2}$. The pinning factor is also a measure of the Fermi-level stabilization at an MSI. The measured value of *S* ~0.5 (Table 2) is indicative of partial Fermi-level stabilization and is similar to the value reported for the other two well-studied gate dielectrics, HfO_2_ and La_2_O_3_ [25]. The moderate value of this parameter indicates that it plays a role in defining the band alignment and in the stoppage of charge transfer (low leakage currents). Furthermore, the *S* value implies that weak pinning probably originated due to the interfacial bonding of moderate/average quality. LaGdO_3_/Si is an interacting heterostructure and, hence, an interfacial layer (IL) consisting of a metal silicate layer, and an SiO*_x_* layer is expected to form between the dielectric and substrate channel during the film formation process. The interface state density and the silicon channel mobility are greatly influenced by the chemical composition and the electronic structure of this IL.

**Figure 11.**
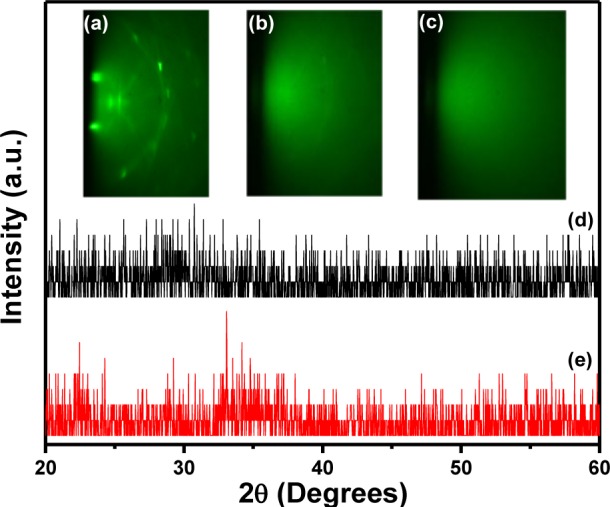
The *in situ* evolution of structure less RHEED patterns during LaGdO_3_ film growth: (**a**) on Si (100) substrate; (**b**) after deposition by one laser ablation shot (layer thickness ~2 Å); (**c**) after completion of deposition (layer thickness: ~30 nm**)**. The XRD spectra of amorphous LaGdO_3_ ultra-thin films of thicknesses (**d**) ~4 nm and (**e**) ~30 nm on Si (100) substrates. Reproduced by permission of ECS–The Electrochemical Society [24].

**Figure 12.**
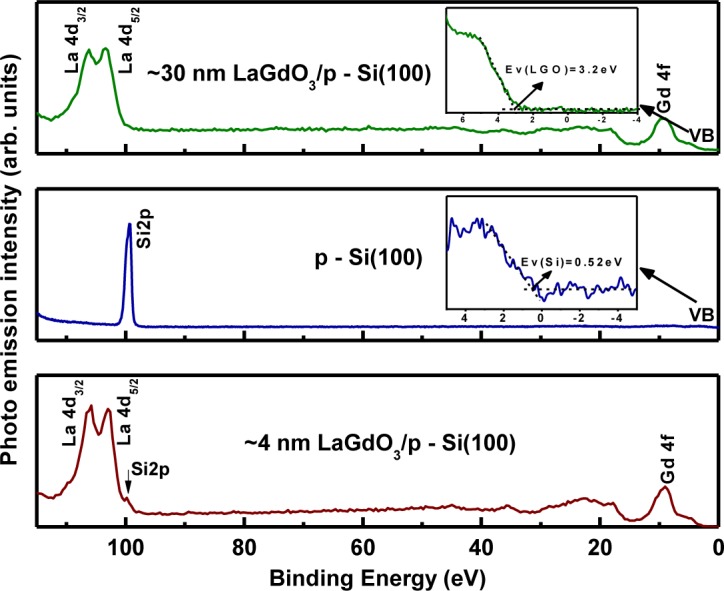
The XPS spectra of shallow core levels and valence band in the ~4-nm LaGdO_3_/Si ultra-thin film, HF-last dipped p-Si (100) and the ~30-nm LaGdO_3_/Si thin film (bulk oxide). The high-resolution scans of the silicon and LaGdO_3_ valence band regions are shown in the inset. Reprinted with permission from [19]. Copyright 2012 AIP Publishing LLC.

Before a particular high-k material is selected for CMOS device applications along with Si channel, it is very important to know the bandgap and the band offsets between it and the semiconductor channel. Such a study will help to find out how the bands are lined up and whether there is sufficient offset (preferably ≥1.5 eV) in the valence band (*∆E*_v_*)* and conduction band (*∆E*_c_*)* to pursue the high-k application. Kraut procedure [26] is a widely used method to estimate the valence band offset (Δ*E*_v_) in a semiconductor-oxide heterojunction with a high degree of accuracy and involves concurrent measurements of the core level and valence band spectra in the bulk semiconductor, bulk oxide and ultra-thin heterostructure using high-resolution XPS. The conduction band offset (Δ*E*_c_) can be derived by using the energy bandgap values of the semiconductor and the oxide materials. For this purpose, bulk oxide (~30 nm-thick) film and ultra-thin film (~4 nm-thick) of LaGdO_3_ were fabricated on an HF-last Si (100) substrate by PLD. Figure 11a shows the RHEED pattern along the (100) azimuth of silicon with visible Kikuchi lines in a high vacuum (2 × 10^−6^ Torr) before commencement of LaGdO_3_ deposition. The *in situ* evolution of featureless RHEED images corresponding to the disordered phase during the growth of LaGdO_3_ at 300 °C, 250 mJ laser pulse energy and ~1 mTorr oxygen partial pressure are given in Figure 11b,c. The XRD spectra of these LaGdO_3_/Si films are depicted in Figure 11d,e, respectively. The absence of any Bragg peaks revealed the amorphous structure of the as-grown films. The valence band maximum of silicon and LaGdO_3_ in the bulk form with respect to the Fermi level was determined by extrapolating the leading edge of the valence band spectrum to the base line and finding the intersection point. The binding energy difference between the appropriate shallow core peaks (La 4*d*_5/2_ and Si 2*p*) and the valence band edge was measured for these two samples and referenced with the core level binding energies of the ultra-thin heterostructure in order to calculate the valence band barrier height (Figure 12), as formulated below [27]:

$$\Delta E_{v}={\left(E_{\mathrm{Si}2p}-E_{v}\right)}_{\text{Bulk}\;\mathrm{Si}}-{\left(E_{\mathrm{La}4d_{5/2}}-E_{v}\right)}_{\text{Bulk}\;\mathrm{La}\mathrm{Gd}O_{3}}-{\left(E_{\mathrm{Si}2p}-E_{\mathrm{La}4d_{5/2}}\right)}_{\mathrm{La}\mathrm{Gd}O_{3}/\mathrm{Si}}$$ (9)

**Figure 13.**
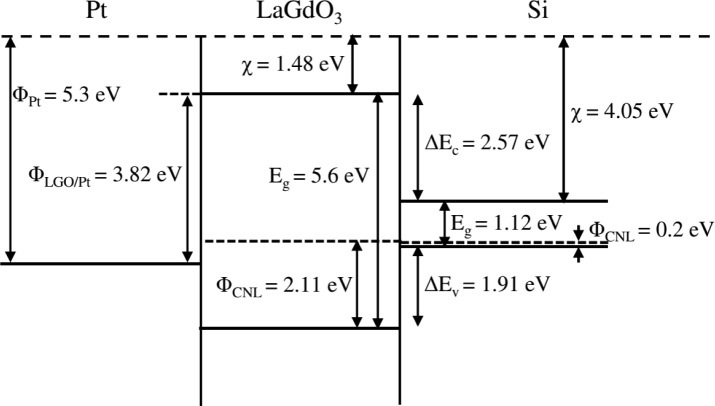
The energy band match-up diagram for the Pt/LaGdO_3_/p-Si MOS device in the flat-band condition.

The energy separation between the Si2*p* centroid and the leading valence band edge in HF-last dipped *p*-Si was estimated to be 98.96 ± 0.05 eV [28]. The corresponding value between the La 4*d*_5/2_ centroid and the leading edge of valence band for bulk LaGdO_3_ film was 100.2 ± 0.05 eV. The binding energy difference between the La 4*d*_5/2_ and Si2*p* core levels in the ultra-thin film was estimated to be 3.15 ± 0.05 eV. Substituting these values in Equation (9), we determined the valence band offset to be 1.91 ± 0.15 eV. Having the values of the average energy bandgap (5.6 eV) from optical transmission spectroscopy and the valence band offset from XPS analysis, we determined the conduction band offset using the following relation,

$$\Delta E_{c}=E_{g({\text{LaGdO}}_{\text{3}})}-E_{g(\mathrm{Si})}-\Delta E_{v}$$ (10)

where $E_{g(\mathrm{La}\mathrm{Gd}O_{3}(}$ is the energy bandgap of amorphous LaGdO_3_ and *E*_g(Si)_ (1.12 eV) is the corresponding parameter for silicon. These extracted parameters are schematically illustrated in the band lineup diagram in Figure 13. The estimated conduction and valance band discontinuity are high enough to suppress the electron or hole injection into the conduction and valence band of LaGdO_3_ from the Si substrate; thus, effecting higher accumulation capacitance and reduction in the gate oxide leakage, meeting one of the qualification criteria of gate oxides.

The band offset is determined by the energy gaps of the semiconductor channel and the gate oxide and the high-k/Si interface dipole generated as a result of the electron transfer from the electropositive species, La and Gd, across the interface to the SiO*_x_* interlayer or silicon channel. The electron and hole transfer across/between the interface gap states (within the energy bandgap) of the semiconductor and gate oxide tends to align the Fermi level of the semiconductor and their interface gap states, which is defined as the charge neutrality level (CNL). The ideal Schottky barrier height (SBH) model is not applicable for LaGdO_3_, as its *S* ≈ 0.5, and this value predicts an asymmetric band match-up and is observed as depicted in Figure 13. Substituting the estimated values of pinning factor *S*, electron barrier Δ*E*_c_ and the reported value of Φ_CNL_ for Si (0.2 eV) [25] in the modified electron affinity rule, proposed by Cowley and Sze [29],

$$\Delta E_{C}=(\chi_{\mathrm{La}\mathrm{Gd}O_{3}}-\Phi_{\mathrm{CNL},\mathrm{La}\mathrm{Gd}O_{3}})-(\chi_{\mathrm{Si}}-\Phi_{\mathrm{CNL},\mathrm{Si}})+S(\Phi_{\mathrm{CNL},\mathrm{La}\mathrm{Gd}O_{3}}-\Phi_{\mathrm{CNL},\mathrm{Si}})$$ (11)

we have estimated Φ_CNL_ of LaGdO_3_ to be 2.11 ± 0.05 eV above its valence band maximum (VBM). It is worth mentioning that the above expression (Equation (11)) is a simplified form of the original Cowley and Sze relation, in which the extra term corresponding to the space charge in the depletion depth has been discarded, and this was pointed out by Rhoderick and Williams [30] and Dawber *et al*. [31]. This term $V_{1}=2e\varepsilon_{s}N_{D}\delta^{2}/\varepsilon_{i}^{2}$, where ε_s_ ≈ 10ε_0_ and *N*_D_ < 10^18^ cm^−3^ is of the order of 10 meV and is neglected. One may note that the discontinuity/change in the vacuum level has not been considered in our calculations. This vacuum-level discontinuity is not zero, but in other oxide perovskites, Scott *et al*. have shown that it is small (<0.5 eV) [32]. The electron affinity of LaGdO_3_ $\left(\chi_{\mathrm{La}\mathrm{Gd}O_{3}}\right)$ was estimated to be 1.48 eV by subtracting the electron barrier height (Δ*E*_C_) from the electron affinity of Si (χ_Si_). The estimated CNL value of LaGdO_3_ indicates that the conduction state density is higher than the valence state density, and the level is pushed down below the mid-gap of the high-k material and suggests the lower ionic (polar) character of bonding in this material (lower mean electronegativity of the material). The difference in the electronegativities of La and O and Gd and O can be estimated to be greater than 1.7 by using the electronegativity (Pauling scale) values of La (1.1), Gd (1.2) and O (3.44) and suggests polar/ionic bonding between them. The ionicity of this high-k dielectric implies that proper work function tuning of the gate metal may be required for LaGdO_3_-based NMOS (N-type metal-oxide-semiconductor) and PMOS (P-type metal-oxide-semiconductor) devices. A larger electron barrier (2.57 ± 0.15 eV) than the hole barrier (1.91 ± 0.15 eV) indicates that LaGdO_3_ films can keep the Schottky emission low enough. A Type I straddled band is observed between LaGdO_3_ and silicon due to the fact that the CNL of the former lies asymmetrically in the lower half of its energy gap, as reported for La_2_O_3_ and Gd_2_O_3_ [33].

## 5. Amorphous LaGdO_3_-Based High-k Metal Gate Devices with Sub-Nanometer Equivalent Oxide Thickness

Amorphous thin films of LaGdO_3_ were fabricated on two types of H-terminated p-Si substrates (thin films on (111)-oriented Si with ~3–6 Ω·cm resistivity and ultra-thin films on (100)-oriented Si with ~0.1–1 Ω·cm resistivity) by using PLD, as described in the previous section, in order to evaluate the device performance of this new material as gate oxides. The major approaches implemented towards achieving sub-nanometer EOT, with reduced defects (e.g., oxygen vacancies) in the LaGdO_3_ layer and minimized LaGdO_3_/Si interfacial reaction, include: (i) optimization of deposition temperature to a low value of ~300 °C; (ii) control of the time of oxygen introduction into the growth chamber and the duration of the exposure of the bare Si wafer to oxygen ambient; (iii) lower oxygen partial pressure of ~1–2 mTorr during ablation; and (iv) preserving the ultra-thin heterostructures in an inert environment of high purity argon. Platinum electrodes of an area ~2.5 × 10^−5^ cm^2^ and a thickness of about 50 nm were DC sputtered at a power density of ~1 W/cm^2^ through a square metal shadow mask to form Pt/LaGdO_3_/p-Si metal-oxide-semiconductor capacitor (MOSCAP) HKMG devices. The resulting structures were annealed in forming gas at 400 °C for 20 min to reduce the interface trap density.

**Figure 14.**
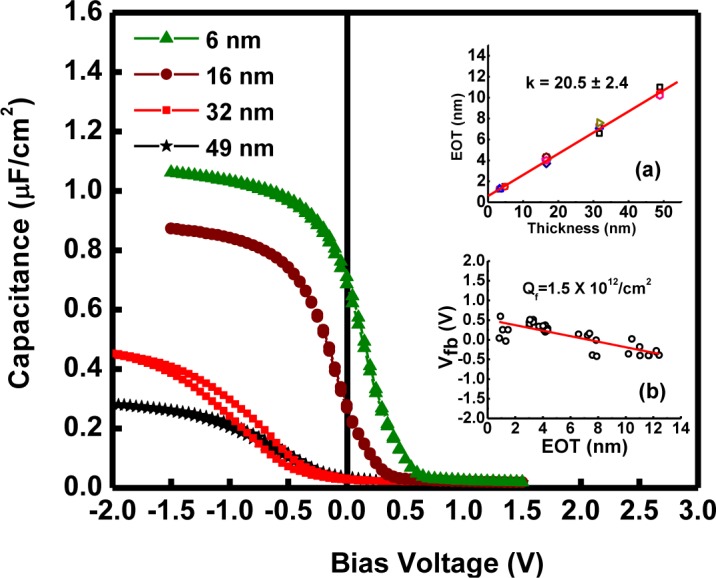
The high-frequency *C-V* characteristics of Pt/LaGdO_3_/Si(111) gate stacks with varying LaGdO_3_ film thicknesses from six to 49 nm. (**a**) The XRR thickness as a function of the equivalent oxide thickness (EOT) of the metal-oxide-silicon (MOS) capacitors to determine the k-value and interfacial layer thickness; (**b**) the flat band voltage *versus* EOT plots to extract the fixed charges. Reprinted with permission from [17]. Copyright 2013 AIP Publishing LLC.

Figure 14 shows the *C-V* characteristics of forming gas passivated n-type MOS devices measured at a frequency of 100 kHz. The dielectric constant (k) was estimated to be 20.5 ± 2.4 from the accumulation capacitance density in terms of the EOT (without quantum mechanical correction) as a function of the XRR (X-ray-reflectometry) physical thickness of the LaGdO_3_ layer on both types of substrates, as depicted in Inset (a) of Figure 14. The intercept on the EOT axis (in the same inset) reveals that there exists an interfacial layer (perhaps of La-Gd silicate) having a thickness of 4.5 ± 1Å with a moderate high-k-value (~8–14 for La silicate [34], much higher than that of SiO_2_) formed, possibly due to the reaction between LaGdO_3_ and Si at the interface. In the majority of the atmosphere/ambient-exposed thicker films with EOT greater than 8 nm, the observed flat-band voltage (*V*_fb_) shift was negative, which is undesirable for CMOS applications and could be due to the presence of positive oxide charges in the form of oxygen vacancies *V*_0_^2+^ [35] and/or due to the incorporation of moisture (which replaces O_2_^−2^ sites with (OH)^−^ in amorphous high-k oxide) [36]. On the other hand, the thinner films with an electrical functional thickness <1.5 nm, which were preserved in an inert argon environment, generally exhibited a positive flat band voltage shift, indicating the presence of negative charges in the form of interstitial oxygen (O*_i_*^2−^) in LaGdO_3_. Despite the small uncertainties in the experimental values, the EOT *versus V*_fb_ plot, as shown in Inset (b) of Figure 14, was found to have a linear fit, indicating that dipoles at the high-k/Si interface are responsible for the observed *V*_fb_ shift, and the fixed charge density, *Q*_f_, estimated from slope of the straight line was 1.5 × 10^12^ /cm^2^.

**Figure 15.**
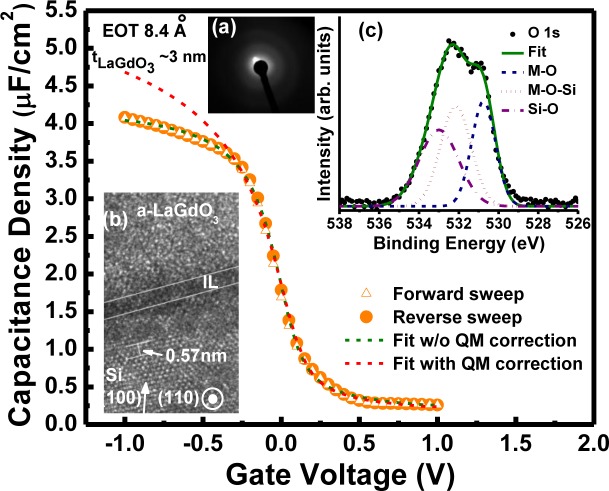
*C-V* plots of Pt/LaGdO_3_/p-Si high-k metal gate devices recorded at 100 kHz, including its Hauser fit with and without quantum mechanical (QM) correction. (**a**) Selective area electron diffraction image of amorphous LaGdO_3_; (**b**) cross-sectional TEM image of the LaGdO_3_/Si heterostructure fabricated at 300 °C; (**c**) *ex situ* O1s XPS spectra from ~8 nm-thick LaGdO_3_ film on Si after Ar+ sputtering etches. Reprinted with permission from [17]. Copyright 2013 AIP Publishing LLC.

Figure 15 shows the unsaturated (4.08 μF/cm^2^ at −1 V) close to ideal *C-V* plots of Pt/LaGdO_3_/p-Si n-MOS devices, with an LaGdO_3_ physical thickness of ~3 nm (grown with better interfacial layer control and kept in an argon atmosphere) measured at 100 kHz. A flat band voltage, *V*_fb_, of ~37 mV, a threshold voltage, *V*_t_, of ~1.11 V, a negligibly small hysteresis of ~2 mV (Δ*V*_fb_ between sweeps) and an interface trap density, *D*_it_, of ~6 × 10^12^ cm^−2^ eV^−1^ were determined from the *C-V* curve. The EOTs estimated from the accumulation capacitance by fitting experimental *C-V* data to the ideal simulation curve with and without quantum mechanical correction by using NCSU CVC program [37] were ~5.4 Å and 8.4 Å, respectively. These values make LaGdO_3_ a promising high-k material that has the potential to enable continued scaling. The cross-sectional image of one of the LaGdO_3_/Si heterostructures with an ~8 nm-thick insulating layer investigated using high-resolution transmission electron microscopy (HRTEM), as shown in Figure 15b, revealed a thin, structureless interlayer of a thickness ~6 Å between the LaGdO_3_ layer and Si substrate, due to the formation of La-Gd silicates (dark layer) and/or silicon oxide (SiO*_x_*), and that is in good agreement with the deduced value from electrical measurements. The absence of any sharp rings or bright spots in the selected area electron diffraction (SAED) pattern of the same LaGdO_3_ layer depicted in Figure 15a indicates the amorphous nature of the PLD-grown high-k layer. The O (oxygen) 1s spectra (Figure 15c) of the LaGdO_3_ layer were found to be asymmetrical with a much wider line width (FWHM) of ~3.2 eV in comparison with that of the symmetrical O1s spectra of pure SiO_2_ (a narrower FWHM of ~1.8 eV) [38], indicating possible bonding of oxygen to La and Gd in addition to Si in the interlayer. Multiple oxygen bonding states were recognized by Gaussian deconvolution of the O1s spectra into three components corresponding to three different chemical compositions (*viz*. M (metal) –O–M (530.75 eV, FWHM ~1.4 eV), M–O–Si (532.15 eV, FWHM ~1.8 eV) and Si–O–Si (533 eV, FWHM ~2.3 eV) [39]) and validated the findings from cross-sectional HRTEM measurements. The nonstoichiometric interfacial layer formation is beneficial in two ways: (i) metal silicate has a higher permittivity [34] than SiO*_x_* to boost the sub-nanometer EOT scaling; and (ii) it causes less carrier (remote coulomb) scattering and mobility reduction [40].

**Figure 16.**
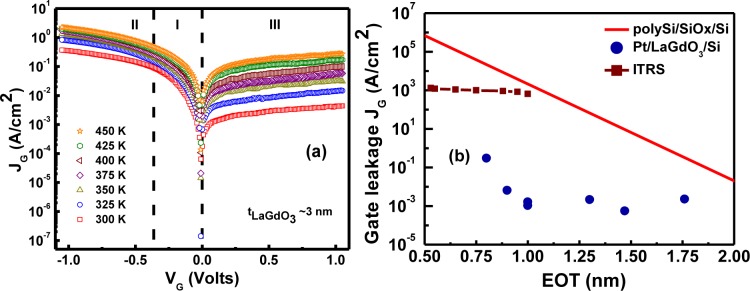
(**a**) The temperature-dependent leakage current density (*J*_G_) *versus* the gate voltage (*V*_G_) characteristics of a Pt/LaGdO_3_/Si(100) n-MOS device; (**b**) comparison of the leakage current densities of various LaGdO_3_ ultra-thin gate stacks with EOT ≤ 1.8 nm (without quantum corrections) with International Technology Roadmap for Semiconductors (ITRS) requirements and thermal SiO_2_ films. Reprinted with permission from [17]. Copyright 2013 AIP Publishing LLC.

Temperature-dependent gate leakage current characteristics were measured for MOS devices with 5.4 Å (with quantum mechanical corrections) and 8.4 Å (without quantum mechanical corrections) EOTs in the 300–450 K temperature range in order to study their current conduction mechanism(s) and device reliability and are illustrated in Figure 16a. A low leakage current density of 0.3 A/cm^2^ (at the sub-nanometer EOT level) was observed at accumulation at an applied gate voltage of 1 V below the flat band voltage (*V*_G_ = *V*_fb_ – 1) (−3.5 MV/cm). The current transport mechanism was found to be Schottky emission dominated at low fields (Region I) and quantum mechanical tunneling at high fields (Region II) during negative gate bias. Positive gate bias current was determined to be a combination of Schottky emission and trap-assisted tunneling (Region III) [17]. Gate leakage current densities of various LaGdO_3_ ultra-thin films on Si as a function of their respective EOTs and their comparison with ITRS limits [3] and classical SiO_2_/Si heterostructures [41] are depicted in Figure 16b. Leakage current densities at −1 V were low and were in the range from 2.3 × 10^−3^ to 0.35 A/cm^2^ for films with EOT from 1.8 to 0.8 nm, and all these values are lower by at least four or more decades in comparison with the stringent ITRS requirements and thermal SiO_2_ films, which validates the charge storage capabilities and, consequently, the applicability of this new material in future technology nodes.

## 6. Long n-Channel LaGdO_3_ Metal-Oxide-Semiconductor Field Effect Transistors (MOSFETs)

**Figure 17.**
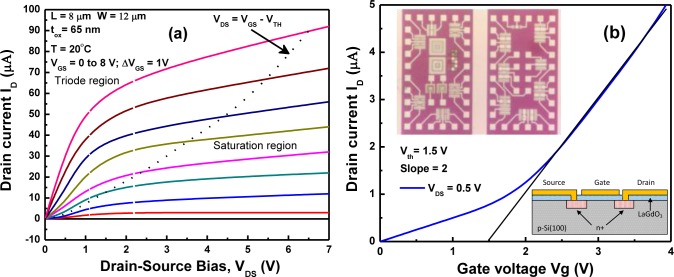
(**a**) Drain current I_DS_ *vs.* drain to source voltage V_DS_ characteristics for LaGdO_3_ gated n-metal-oxide field effect transistor (n-MOSFET) for various gate voltages; (**b**) sub-threshold *I*_D_-*V*_GS_ characteristic for an n-MOSFET device. The inset shows the layout of MOSFET structures on the p-Si wafer with gate lengths between 5 and 50 μm (top) and their schematic layout (bottom).

Encouraging transistor behavior was achieved with high-k LaGdO_3_ thin films as the gate oxide [42]. Figure 17a shows the drain current characteristics of the n-MOSFET with a 65 nm-thick LaGdO_3_ gate-oxide layer, a channel length (*L*) of 8 μm and a channel width (*W*) of 12 μm at room temperature for different gate voltages. The drain current, I_D_, varied from 2 μA to 90 μA with gate voltage variation from its sub-threshold characteristics (Figure 17b) at a constant drain voltage, *V*_D_, of 0.5 V by extrapolating the linear region down to the voltage axis. The transconductance, *g*_m_, evaluated from the slope of this plot was 2 μS. The layout of the MOSFET structures with gate lengths between 5 and 50 μm and their device cross-sections are shown in the insets (top and bottom, respectively) of Figure 17b.

## 7. Amorphous LaGdO_3_-Based High-Density Metal-Insulator-Metal Capacitors with Sub-Nanometer Capacitance Equivalent Thickness

**Figure 18.**
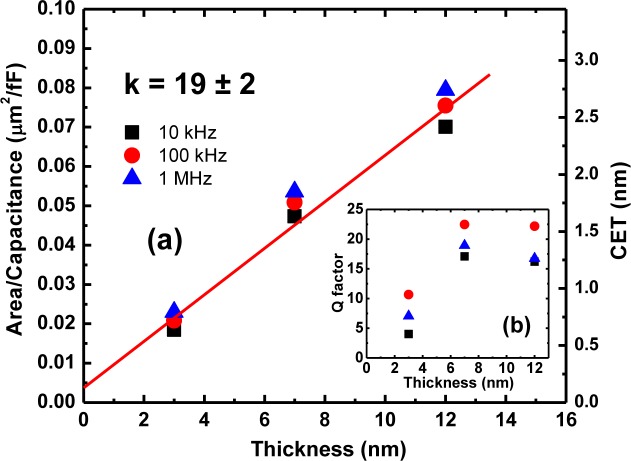
(**a**) The inverse of the capacitance density, capacitance equivalent thickness (CET); and (**b**) the Q-factor for several AC signal frequencies at zero bias field for LaGdO_3_ thin films with different thicknesses. Reprinted with permission from [43]. Copyright 2013 AIP Publishing LLC.

In this section, the characteristics of planar MIM mono-dielectric layer stacks fabricated using pulsed laser deposited thin films of high-k dielectric LaGdO_3_ are presented in order to evaluate the performance of this new material for more demanding MIM capacitor applications, such as radio frequency (RF) coupling and bypass capacitors in oscillators and resonator circuits, filter and analog capacitors in analog/mixed-signal (AMS) circuits, decoupling capacitors for microprocessors (MPUs), as well as storage capacitors in DRAM and embedded DRAM (eDRAM)/logic devices. Figure 18 shows the inverse of capacitance density and capacitance equivalent thickness (CET) of Pt/LaGdO_3_/Pt structures at zero bias field as a function of dielectric layer thickness for several AC signal frequencies. The dielectric constant (k) value was determined to be 19 ± 2 from the slope of the straight line fit in Figure 18a, and it can also be concluded from the intercept on the ordinate that there exists a parasitic interfacial layer of unknown composition having a thickness of 1.5 ± 0.3 Å, developed possibly due to reaction between LaGdO_3_ and platinum at the interface. The capacitance densities achieved were promisingly high enough (~19–21 fF/μm^2^ at a CET of 1.75 nm) for MIM device applications and were found to remain nearly constant throughout the applied bias voltage range. *C-V* characteristics recorded at 100 kHz indicate a systematic capacitance scaling with dielectric layer thickness, as illustrated in Figure 18a by the increase in capacitance density from ~13.3 to 43.5 fF/μm^2^ with a reduction in CET from ~2.6 to 0.66 nm, respectively, at zero bias voltage. The lowest electrical functional thickness achieved in the present study is ~0.66 nm, and this capacitor has the highest dielectric losses (*Q* = 7.05).

**Figure 19.**
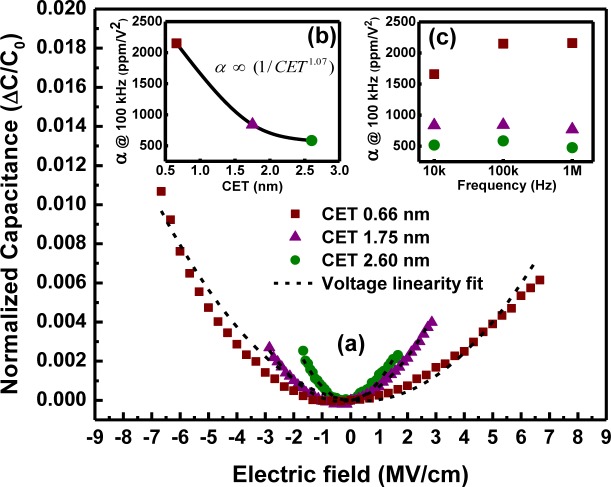
(**a**) The normalized capacitance as a function of applied electric field and voltage linearity fit for various LaGdO_3_ film thicknesses measured at 100 kHz. Variation in the voltage coefficient of capacitance as a function of (**b**) CET at 100 kHz and (**c**) the AC drive signal frequency. Reprinted with permission from [43]. Copyright 2013 AIP Publishing LLC.

**Figure 20.**
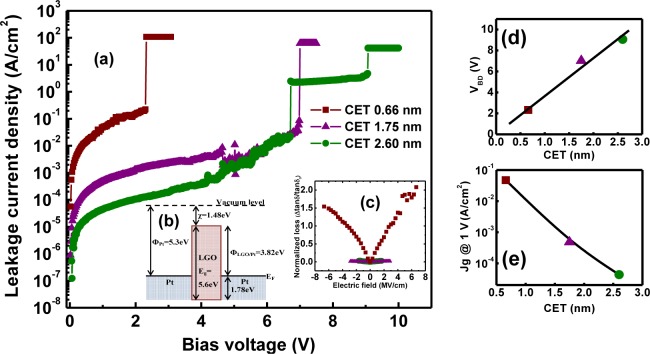
(**a**) The thickness dependence of the leakage current density-bias voltage plots of Pt/LaGdO_3_/Pt MIM capacitors; (**b**) the energy band match-up diagram of the Pt/LaGdO_3_/Pt MIM structure under the zero bias condition showing the larger LaGdO_3_/Pt Schottky barrier height; (**c**) the normalized loss tangent as a function of the applied electric field; (**d**) the linear evolution in breakdown voltage with CET; (**e**) the leakage current density at a 1 V bias voltage for various CETs. Reprinted with permission from [43]. Copyright 2013 AIP Publishing LLC.

The voltage linearity, a key parameter that an alternative high-k linear dielectric candidate for MIM applications must have, can be verified by simulating the experimental *C-V* plots with the quadratic law [44]:

$$\frac{\Delta C}{C_{0}}=\frac{C_{v}-C_{0}}{C_{0}}=\alpha V^{2}+\beta V$$ (12)

where *V* is the applied voltage, *C*_v_ and *C*_0_ are the capacitance at a specific applied voltage and at zero voltage, respectively, α and β are the quadratic and liner voltage coefficients, respectively, expressed in units of parts per million (ppm)/V^2^ and ppm/V, respectively, and Δ*C*/*C*_0_ is the normalized capacitance. α, also called the voltage coefficient of capacitance (VCC), is a more relevant quantity to be minimized, and its value should be maintained within 100–1000 ppm/V^2^ for RF and AMS device applications [3], respectively. As shown in Figure 19a, the normalized capacitance following a positive parabolic path (positive αvalues) with bias voltage may be due to the high degree of electric field polarization and carrier injections [45,46], and this parameter increases with the reduction in dielectric thickness (electric field enhancement) in MIM capacitors, as shown in Figure 19b. VCC increased from 584 ± 0.15 to 2150 ± 3 ppm/V^2^ with a decrease in CET from ~2.6 to 0.66 nm. The thickness dependency of VCC is considered an inherent property and is found to obey the relation α ∝ (1/*t^r^*), where *t*, the high-k film thickness, is proportional to CET. In this study, the estimated value of the exponent “*r*” was small and nearly equal to one [47,48] and shows that LaGdO_3_-based MIM capacitor structures can be down scaled more efficiently (in terms of CET) with lesser voltage linearity reduction in comparison with other high-k materials of larger “*r*” [49]. Normalized capacitances modeled with the voltage linearity law given by Equation (12) are represented by dashed lines in Figure 19a. The observed nonlinearities (non-symmetry) in the *C-V* characteristics for positive and negative bias voltages (large β) may have originated possibly due to the presence of oxygen vacancies, non-linearities of the metal-oxygen bond polarizability, trap sites at the vicinity of the metal-dielectric interface, *etc.* [50,51], and can be improved by post deposition annealing in oxygen/nitrogen plasma, ultraviolet (UV) or ozone environments [52] at temperatures compatible with the back end of line (BEOL) process. It is worth noting that experimentally determined VCC and capacitance densities fit with the theoretical plot reported by Blonkowski *et al.* [44], and the quadratic electric field coefficient, α_E_, for LaGdO_3_ was found to be in the range of (1–4) × 10^−19^ m^2^/V^2^. The variation in α with AC drive frequency for various CETs is depicted in Figure 19c and shows a weak rise in α with decreasing frequency for capacitors with CET of 1.75 and 2.6 nm. This behavior is possibly due to the lower mobility of the free carriers at higher AC signal frequencies, which, in turn, results in a larger relaxation time and a smaller quadratic capacitance variation [50]. At very high frequencies (above 1 MHz), this extrinsic contribution to VCC ${(\alpha }_{E}^{\text{ext}})$ is expected to keep lowering, whereas the thickness-independent intrinsic contribution ${(\alpha }_{E}^{\text{int}})$ from the bulk-effect properties of the films remains steady ${(\alpha }_{E}=\alpha_{E}^{\text{ext}}+\alpha_{E}^{\text{int}})$ [53]. However, the MIM structure with a sub-nanometer CET of ~0.66 nm showed the opposite trend. This behavior can be explained by considering the fact that for thinner layers (~3 nm), the time for the carriers to cross the high-k dielectric medium is smaller and, consequently, the frequency of extrinsic free carrier contribution to the dielectric relaxation must be larger than that for the other two thicker layers. This feature can be further confirmed if one assumes space-charge-relaxation as the origin of the extrinsic contribution, since the cut-off frequency of the relaxation varies in proportion with the conductivity in that case. A higher conductivity (leakage current density of 0.1 A/cm^2^ at 1.5 V (Figure 20)) and a quality factor (Q) of ~7 at zero applied bias (Figure 19b) are obtained for this MIM capacitor, and correspondingly, a higher capacitance variation is observed (α = 2,150 ppm/V^2^ at 100 kHz) with respect to the other two of the higher CETs. The extrinsic carrier relaxation maximizes at higher frequencies, and hence, the net quadratic voltage coefficient/permittivity gets enhanced. The percentage variation in the dielectric constant is displayed in Table 3. It is worth mentioning that all the ultra-thin MIM capacitors investigated have shown a very low capacitance/permittivity variation (<0.6144%) at ± 2 V bias in the 10 kHz–1 MHz frequency range.

Ambient leakage current density-bias voltage characteristics beyond the dielectric breakdown voltage are displayed in Figure 20a for three different thicknesses of the LaGdO_3_ layer. The Schottky barrier height for the symmetric MIM architecture was determined to be ~3.82 eV (Figure 20b), by considering the work function of Pt (Φ = 5.3 eV) and the previously determined energy bandgap (*E*_g_ = 5.6 eV) and electron affinity (χ = 1.48 eV) values of LaGdO_3_. The linear evolution (scaling) in the breakdown voltage with CET is illustrated in Figure 20d. The equivalent breakdown field estimated from the slope of this plot was about ~36 MV/cm, which comes out to be ~6 MV/cm (breakdown field, E*_BD_* = (equivalent field × 3.9) / *k*) by using the dielectric constant of LaGdO_3_ (*k* ~ 22). One may notice that this obtained experimental value is comparable with the theoretical value estimated (5.5 MV/cm) by applying the “square root” rule in accordance with the thermo-chemical/molecular model [54] for the breakdown field $E_{\text{BD}}=29.9\varepsilon_{r}^{-0.55}$. The maximum energy storage density was calculated to be ~40 J/cm^3^, according to the equation $W=(\varepsilon_{\text{0}}\varepsilon_{r}E_{\text{BD}}^{2}/2)$, and this higher figure suggests that LaGdO_3_-based MIM architectures may be of use for energy density capacitor applications in energy storage and power compression devices [55]. At a particular bias voltage, the leakage current density increased with the reduction in LaGdO_3_ film thickness (Figure 20e) as a result of the rise in electric field; analogous to the variation in the quadratic voltage coefficient (Figure 19b). One may note in Figure 20c that the normalized tangent loss increases with applied electric field, and the MIM capacitor with the thinnest LaGdO_3_ layer has the highest conductivity. Leakage current densities recorded at a 1 V bias voltage were 4.3 × 10^−5^, 4.8 × 10^−4^ and 4.7 × 10^−2^ A/cm^2^ for 2.6-, 1.75- and 0.66-nm CETs, respectively.

## 8. Conclusions

The room temperature, perfectly layered B-type monoclinic crystal structure of LaGdO_3_ has been established utilizing the X-ray diffraction method, and its structural evolution with temperature has been probed by Raman spectroscopy studies. This high-k material has been identified as a potential high-k candidate through the investigation of the temperature and frequency-dependent dielectric properties, the leakage conduction mechanism of polycrystalline ceramics and the physical, optical, XPS and electrical characterizations of amorphous thin films. Encouraging figures were obtained for the complex refractive index, the dispersion parameters of the index of refraction, the energy bandgap, the band offsets of the LaGdO_3_/Si heterostructure, the interface parameter and the permittivity. The interfacial layer thickness in LaGdO_3_/Si ultra-thin heterostructures was minimized by controlling the pulsed laser deposition parameters and the associated conditions to achieve sub-nanometer EOTs in nMOS HKMG stacks. Studies on ultra-thin LaGdO_3_ layer-based planar MIM capacitor architectures revealed the applicability of this new material in next generation radio frequency, analog/mixed-signal and dynamic random access memory devices. In summary, this comprehensive work shows that LaGdO_3_ meets the major alternative high-k material challenges, and this new electronic device material has been demonstrated as a gate dielectric of great potential.

## Figures and Tables

**Table 1.** The double power law parameters obtained by fitting the experimental data. Reprinted with permission from [15]. Copyright 2012 AIP Publishing LLC.

Temperature (K)	σ_dc_ (S cm^−1^)	*n*_1_	*n*_2_
250	8.57 × 10^−11^	0.947	2
300	1.11 × 10^−09^	0.960	1.996
350	1.94 × 10^−09^	0.837	1.858
400	3.51 × 10^−09^	0.774	1.993
450	1.22 × 10^−08^	0.638	1.577
500	1.31 × 10^−07^	0.566	1.584

**Table 2.** Dispersion parameters of the index of refraction of amorphous LaGdO_3_ samples estimated by modeling the experimental data with the Sellmeier formula. Reprinted with permission from [19]. Copyright 2012 AIP Publishing LLC.

Dispersion Parameters	Film Thickness (nm)		
	725	350	170
Refractive index *n* @ (550 nm)	2.073	2.083	2.105
Average oscillator strength *S*_0_ (×10^14^ m^−2^)	1.67	1.66	1.5
Average oscillator position λ_0_ (nm)	136.36	137.18	146.13
Oscillator peak energy *E*_0_ (eV)	9.10	9.04	8.49
Refractive index dispersion parameter *E*_0_/*S*_0_ (×10^−14^ eV·m^2^)	5.44	5.44	5.65
Dispersion energy *E*_d_ (eV)	28.29	28.29	27.32
Measure of dispersion *A* (×10^−16^ m^2^)	59.88	60.24	66.67
Long wavelength refractive index *n*_∞_	2.026	2.031	2.050
Electronic dielectric constant ε_∞_	4.104	4.124	4.2
Mean polarizability α_e_ (Å^3^)	9.525	9.553	9.670
Pinning factor *S*	0.509	0.506	0.494
Interface parameter *S*′	1.155	1.149	1.121

**Table 3.** Percentage of variation in the k-value with AC signal frequency for different CETs. Reprinted with permission from [43]. Copyright 2013 AIP Publishing LLC.

CET (nm)		0.66	1.75	2.6
Variation in k-value ((*C*_2V_ − *C*_0V_)/*C*_0V_)(%)	10 kHz	0.1361	0.4009	0.2486
	100 kHz	0.6144	0.3986	0.2311
	1 MHz	0.5919	0.3219	0.1132
